# Manipulating and measuring single atoms in the Maltese cross geometry

**DOI:** 10.12688/openreseurope.13972.1

**Published:** 2021-09-06

**Authors:** Lorena C. Bianchet, Natalia Alves, Laura Zarraoa, Natalia Bruno, Morgan W. Mitchell

**Affiliations:** 1 ICFO - Institut de Ciències Fotòniques, The Barcelona Institute of Science and Technology, Castelldefels, Barcelona, 08860, Spain; 2 Istituto Nazionale di Ottica (CNR-INO), Largo Enrico Fermi 6, Florence, 50125, Italy; 3 European Laboratory for Non-linear Spectroscopy (LENS), Via nello Carrara 1, 50019 Sesto Fiorentino, Florence, Italy; 4 ICREA - Institució Catalana de Recerca i Estudis Avançats, Barcelona, 08010, Spain

**Keywords:** Single atom, high numerical aperture, optical tweezers, Maltese cross, optical lattice, resonance fluorescence, single quantum emitter, parametric excitation

## Abstract

**Background:** Optical microtraps at the focus of high numerical aperture (high-NA) imaging systems enable efficient collection, trapping, detection and manipulation of individual neutral atoms for quantum technology and studies of optical physics associated with super- and sub-radiant states.  The recently developed “Maltese cross” geometry (MCG) atom trap uses four in-vacuum lenses to achieve four-directional high-NA optical coupling to single trapped atoms and small atomic arrays. This article presents the first extensive characterisation of atomic behaviour in a MCG atom trap.

**Methods:** We employ a MCG system optimised for high coupling efficiency and characterise the resulting properties of the trap and trapped atoms.  Using current best practices, we measure occupancy, loading rate, lifetime, temperature, fluorescence anti-bunching and trap frequencies. We also use the four-directional access to implement a new method to map the spatial distribution of collection efficiency from high-NA optics:  we use the two on-trap-axis lenses to produce a 1D optical lattice, the sites of which are stochastically filled and emptied by the trap loading process. The two off-trap-axis lenses are used for imaging and single-mode collection.  Correlations of single-mode and imaging fluorescence signals are then used to map the single-mode collection efficiency.

**Results: **We observe trap characteristics comparable to what has been reported for single-atom traps with one- or two-lens optical systems. The collection efficiency distribution in the axial and transverse directions is directly observed to be in agreement with expected collection efficiency distribution from Gaussian beam optics.

**Conclusions: **The multi-directional high-NA access provided by the Maltese cross geometry enables complex manipulations and measurements not possible in geometries  with fewer  directions of  access,  and can  be  achieved  while  preserving other trap characteristics such as lifetime, temperature, and trap size.

## Plain language summary

In this article we report measurements performed on individual atoms held in place by focused laser beams. The atoms are in vacuum, which prevents them from coming into contact with other atoms or molecules. By using four lenses, placed around the equator of a sphere centered on the atom, we are able to collect light emitted by the atom in different directions, and also to illuminate the atom from different directions simultaneously. One of the main aims of this work is to measure the characteristics of an atom trapped in this kind of four-lens system. We measure the rate at which atoms are collected into the trap, how long they remain before leaving, the average kinetic energy of the atom, the emission pattern that evidences the presence of the atom in the trap and a measure of the time correlations of the emitted photons. The results show that in the system with four lenses, the atom is as cold and well-localized as in one- and two-lens systems previously characterized. To demonstrate the possibilities created by having lenses along four directions, we send laser light at the atom from two opposing directions, while also collecting light emitted from the atom along three directions. By sending light along two opposing directions, we create many small traps at which the atom can be immobilized. This allows us to study how well the lenses can collect light from different locations, and in particular demonstrates the ability to selectively collect light from some locations rather than others.

## Introduction

Optical microtraps at the focus of high numerical aperture (high-NA) imaging systems enable efficient collection, trapping, detection and manipulation of individual neutral atoms ^1– 5^ and molecules ^6^. These capabilities are exploited in several active topics in quantum optics and quantum technology, including strong single-atom effects on traveling-wave beams ^7– 12^, higher-order interference of atoms ^13– 15^ and Rydberg-atom-based quantum information processing ^16^ and quantum simulation ^17, 18^. High-NA trapping systems may also enable strong modifications to radiation physics associated with sub-radiant states ^19– 22^.

**Figure 1.**
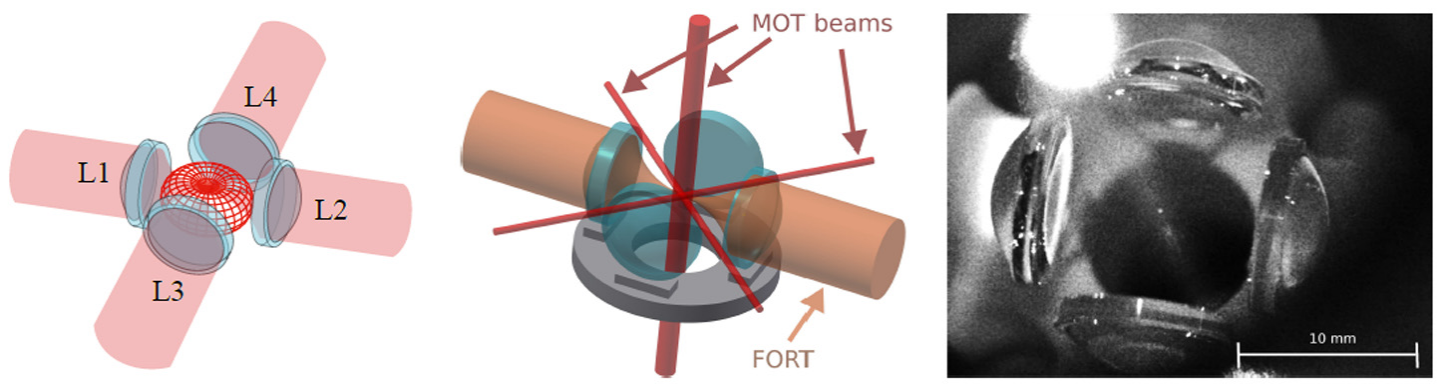
Illustration of the Maltese cross geometry, in which four high-numerical-aperture lenses L1 to L4 are arranged along the cardinal axes. Left: Red meshed structure at center shows the angular distribution of radiation from a vertically-polarized electric dipole transition. The emission is strongest around the equator and thus efficiently collected by the four lenses. Center: In addition to collecting light efficiently, the in-vacuum aspheric lenses can be used for strongly-focused illumination, and to efficiently produce a far-off-resonance trap (FORT). The in-vacuum lenses leave little open solid angle in the equator for magneto-optical trap (MOT) beams, which are necessarily much smaller than in a typical MOT. Right: image of the four-lens assembly used in this work. The small bright spot at the centre of the lens arrangement is the MOT. Large bright spot at the upper left is scattering of the vertically-propagating MOT beams from the vacuum chamber window.

The earliest experiments with neutral-atom microtraps employed large vacuum systems and custom-designed optics ^23^. More recent works have employed high-NA aspheric lenses ^24^ in smaller vacuum systems, which has enabled experiments with high-NA optical access from two ^8, 25^ and four ^26– 29^ directions. The four-lens arrangement is known as the Maltese cross geometry (MCG) when the lenses are placed on the cardinal directions, as illustrated in Figure 1.

**Figure 2.**
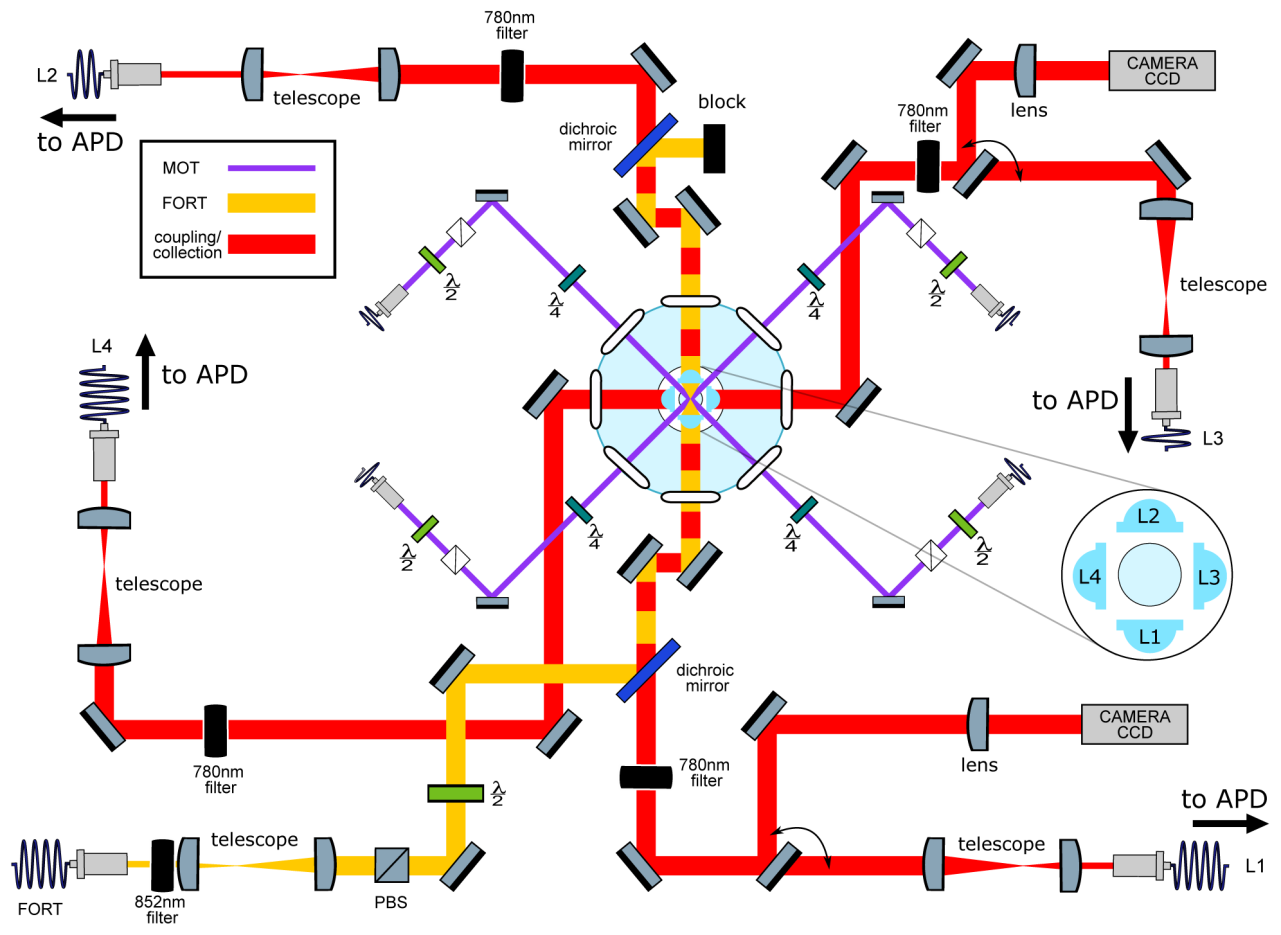
Main elements of the optical setup. Left: Schematic of optical systems. Except when indicated otherwise, all elements lie in a horizontal plane intersecting the trap center. In light blue: Four aspheric lenses (lens numbers L1-L4 are indicated in the inset) are located symmetrically around the geometric center of a “spherical octagon” vacuum enclosure with eight anti-reflection-coated windows. In purple: magneto-optical trap (MOT) beams, which pass through the gaps between the lenses; vertically-directed MOT beams passing through the trap center are not shown. In yellow: far-off-resonance trap (FORT) beam, which is focused by L1 and re-collimated by L2. The beam-block shown in the image can be replaced with a mirror to retro-reflect the beam and produce a 1D lattice, as described in section **Collection-efficiency mapping using stochastic loading**. In red: Beams focused by the in-vacuum lenses for coupling light to and from the single atom. Red/yellow dashing: Coupling beams leading to L1 and L2 are combined on dichroic mirrors with the FORT beam, resulting in coaxial propagation. All four lenses can be used for fluorescence collection, which is either collected with a fiber and sent to an avalanche photodiode detector (APD) or sent to a charge-coupled device (CCD) camera in the case of L1 and L3. Diagram symbols: PBS: polarizing beamsplitter, *λ/*2 ( *λ/*4): half (quarter) waveplate, filter: bandpass filters centered in the specified wavelength.

The MCG can increase the total solid angle coupled to the atom ^28^, and makes possible the measurement of coherent, large-momentum-transfer scattering processes in disordered ensembles ^30^ and in atomic arrays ^19^, for which strong sub-radiant effects are predicted. The right-angle geometry is also predicted to enhance and modify the observable quantum correlations in resonance fluorescence ^31^. The MCG geometry also imposes constraints not present in traps using one or two lenses. Most immediately, the direct (as opposed to through-lens) optical access is greatly reduced in the plane of the four lenses. Forming a magneto-optical trap (MOT) then either requires reduced NA ^32^, a non-orthogonal beam geometry ^8^ or a sub-mm beam diameter ^26^. In the system described here, we use four in-vacuum high-NA aspheric lenses, and pass sub-mm MOT beams through the small gaps between them, as shown in Figure 2. This approach leads to greatly reduced MOT volume and atom number ^28^. At the same time, the use of simple aspheric lenses, rather than multi-element objectives, implies a small diffraction-limited focal region of ∼ 30 µm diameter, and also different focal lengths for the 780nm fluorescence wavelength versus the 852nm far-off-resonance trap (FORT) wavelength ^28^. While physical-optics simulations in Zemax, described in 28, indicate that it should still be possible to achieve diffraction-limited spot sizes for four fluorescence-coupling beams and the FORT beams producing the trap, the tolerances are reduced relative to lens geometries that only couple from one or two directions. These potential perturbations to the cooling and trapping components of the system motivate a characterization of the MCG system’s capacity to produce and optically couple to cold, well-localized trapped atoms and atomic arrays.

In this article we check that the incorporation of a third and fourth lens has not degraded the optical system’s ability to trap, cool and couple light to single atoms. To this end, we measure occupancy, loading rate, lifetime, temperature, fluorescence anti-bunching and trap frequencies using current best practices. To our knowledge, these characterizations have not been reported for any four-lens trapping system. We observe trap characteristics comparable to what has been reported for single-atom traps with one- or two-lens optical systems, thus confirming that the MCG provides significant advantages in flexibility and total degree of coupling, without sacrificing other desirable features. With this utilitarian objective completed, we explore the new geometry’s capabilities and characterize its spatial collection. To do so, we employ each of the four lenses in a different way in a single experiment, to produce an optical lattice, perform single-mode light collection both on-axis and off-axis, and also collect off-axis light onto an imaging sensor. This allows us to observe the stochastic loading and unloading of an optical lattice with three distinct collection modalities, and to quantify spatio-temporal correlations among them. In this way we are able to map the spatial distribution of collection efficiency of both on-axis and off-axis single-mode collection.

The article is organized as follows: In section **System description** we describe the experimental system, including MOT, far-off-resonance trap (FORT), and atomic fluorescence collection. In section **Trap characterization** we characterize the trap lifetime, temperature, and trap frequencies. In section **Collection-efficiency mapping using stochastic loading**. we measure the spatial distribution of collection efficiency for one axial and one transverse high-NA collection lens, using the correlation of fluorescence seen in single-mode collection with that seen in imaging detection.

## System description

The system employs a small MOT to collect and cool a cloud of rubidium-87 ( ^87^Rb) atoms from background vapor in an ultra-high vacuum enclosure, and load them into a FORT located within the MOT volume. The MOT and FORT centers are co-located at the center of a system of four high-NA lenses (NA=0.5) along the cardinal axes. A detailed description of the high-NA optics, assembly and characterization is given in 28. Here we describe other critical elements of the trapping and cooling system, which is illustrated in Figure 2.

### MOT

A small MOT is formed by six counter-propagating beams along three orthogonal axes in the standard configuration. The Repumper light is on resonance with the ^87^Rb D _2_ transition 5 *S*_1 */*2_, *F* = 1 → 5 *P*_3 */*2_, $F^{\prime }$ = 2, where *F* ( $F^{\prime }$ ) indicates the hyperfine level of the 5 *S*_1 */*2_ ground state (5 *P*_3 */*2_ excited state). Cooler light is red-detuned from the 5 *S*_1 */*2_, *F* = 2 → 5 *P*_3 */*2_, $F^{\prime }$ = 3 transition by 6Γ _0_, where Γ _0_ = 2 *π* × 6.06 MHz is the D _2_ natural linewidth. To pass cleanly between the 1.2 mm gaps separating the lenses, the horizontally-directed beams are of 0.7mm diameter, whereas the vertical beams are of 2.0mm diameter. Horizontal and vertical cooler beams have powers of 20 µW and 162 µW, respectively. Repump light of 150 µW is sent only in the downward vertical direction, to minimize scattered light. For the single-atom experiments described below, a MOT gradient of 3.8 G cm ^−1^ is used, to reduce the number of MOT atoms and resulting background fluorescence. In this scenario, we produce a ≈ 50 µm diameter cloud of cold atoms to be superimposed with the FORT described in section **FORT**.

### FORT

The FORT is produced by a linearly-polarized 852 nm beam with a power of 7 mW and a beam waist of 1.85 mm at the aspheric lens position. The laser used to produce this beam is a distributed feedback (DFB) laser (Toptica Eagleyard EYPDFB0852) stabilized to the 6 *S*_1 */*2_, *F* = 4 → 6 *P*_3 */*2_, $F^{\prime }$ = 5 Cs D _2_ transition by modulation transfer spectroscopy (MTS) ^33^. The wavelength-scale size of the waist at focus creates a dipole micro-trap of few-µm ^3^ volume. In the presence of cooler light, e.g. if the MOT is on, light-assisted collisions (LACs) ^23^ rapidly remove any pairs of atoms in this small volume. In practice, this ensures the presence of no more than one atom in the trap. The 852 nm FORT wavelength is sufficiently far from resonance as to produce little scattering by the trapped atom, yet close enough that a single aspheric lens can be diffraction limited when focusing both it and the spectroscopic wavelengths 780 nm (D _2_) and 795 nm (D _1_). 852 nm also coincides with the Cs D _2_ line, which is convenient for frequency stabilization and atomic filtering. To position the dipole trap midway between the two lenses, a shearing interferometer (SI) is used to measure the beam divergence before the input lens, and after the output lens, and to set the divergences to be equal and opposite. The same SI is used in this symmetric condition to check for aberrations. For more details see 28.

Within the Gaussian beam approximation, the FORT potential is


$$U_{\text{FORT}}(r,z)=\beta P_{\text{FORT}}\frac{2}{\pi w^{2}(z)}\exp[-\frac{2r^{2}}{w^{2}(z)}]\;(1)$$

where $r=\sqrt{x^{2}+y^{2}}$ is the transverse radial coordinate, *z* is the axial coordinate, *β* ≈ −6.39 × 10 ^−36^ Jm ^2^W ^−1^ is the ground state light shift coefficient ^34^, *P*_FORT_ is the power of the FORT beam, $w(z)\equiv w_{\text{FORT}}\sqrt{1+z^{2}/z_{R}^{2}}$ where *w*_FORT_ is the FORT beam waist, and $z_{R}\equiv \pi w_{\text{FORT}}^{2}/\lambda_{\text{FORT}}$ is the Rayleigh length.

In most circumstances, the atom’s thermal energy is far less than the trap depth *k _B_T*_atom_ ≪ *U*_0_ ≡ | *U*_FORT_(0, 0)|, where *k _B_* is the Boltzmann constant, and it is thus appropriate to use the harmonic approximation *U*_FORT_( *r*, *z*) ≃ *U*_0_[−1 + 2( *r/w*_FORT_) ^2^ + ( *z/z _R_*) ^2^].

### Fluorescence collection

**Figure 3.**
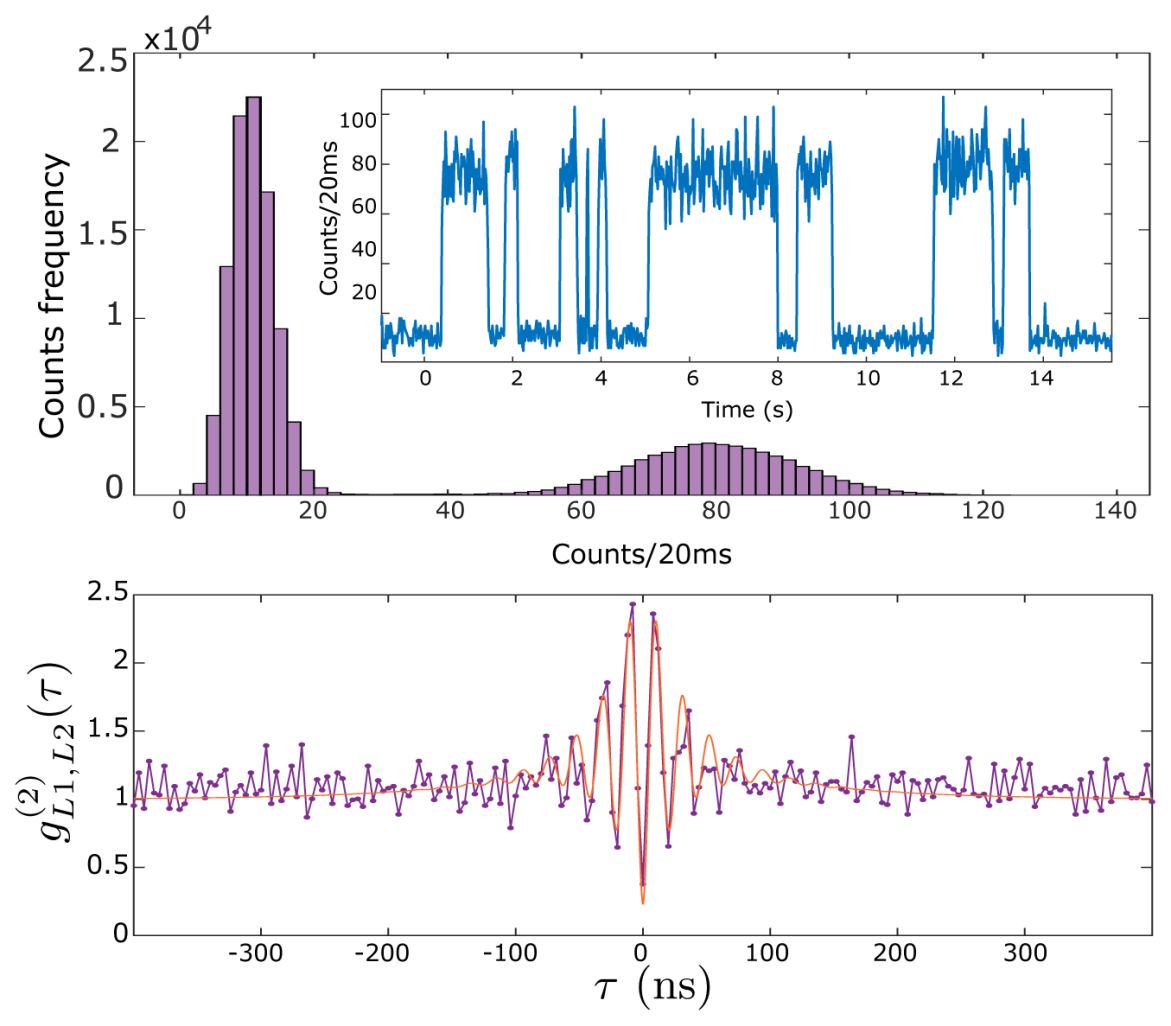
Single-atom resonance fluorescence. Upper plot: time series (inset) and histogram (main graph) of collected fluorescence from L1 as single atoms enter and leave the trap with magneto-optical trap (MOT) and far-off-resonance trap (FORT) in continuous operation, see text for details. Lower plot: normalized cross-correlation $g_{L1,L2}^{\left(2\right)}(\tau )$ between collection channels L1 and L2. Points show data, red curve shows a fit with $g_{L1,L2}^{\left(2\right)}(\tau )\;=\;1-A\exp[-\left|\tau \right|/\tau_{1}]\;\cos({\text{Ω}}^{\prime }\tau )\;+\;B\exp[-\left|\tau \right|/\tau_{2}]$ with fitting parameters ${\text{Ω}}^{\prime }$ = 2 *π* × 47.6 MHz, *τ*_1_ = 28.5 ns, *τ*_2_ = 90.2 ns, *A* = 1.26 and *B* = 0.48. This fit function is a heuristic approximation to the four-level *g* ^(2)^( *τ*) found numerically, for example in 36. See HistogramAndNormalizedCrossCorrelation.csv in *Underlying data* ^35^.

The fluorescence collected by each lens is sent to a different avalanche photodiode detector (APD). Counts in each APD are recorded by an Arduino Due microcontroller and typically binned into 20ms time bins. A representative signal is shown in the inset of the upper plot of Figure 3 (see *Underlying data* ^35^). This shows a random telegraph signal, i.e., stochastic switching between just two signal levels, corresponding to the zero-atom and one-atom conditions. The main figure of the upper plot shows a histogram of the counts of this telegraph signal for a measurement of 2700 s duration. It is clear that counts corresponding to zero atoms are well distinguishable from the counts corresponding to one atom in the trap. Due to LACs, larger atom numbers are not observed. We use this real-time telegraph signal for fine alignment of the collection fibers to the atom. The clear gap in counts allows us to perform sequence measurements triggered by the presence of an atom in the trap. The lower plot in Figure 3 shows the normalized cross-correlation $g_{L1,L2}^{\left(2\right)}$ ( *τ*) of the signals collected via L1 and L2 (see *Underlying data* ^35^). Antibunching, i.e. $g_{L1,L2}^{\left(2\right)}$ (0) < 1, indicates a non-classical photon flux typical of single-emitter systems. The background level $g_{L1,L2}^{\left(2\right)}$ ( *t*), *t* ≫ $\Gamma_{0}^{-1}$ , is due to MOT fluorescence [ 28, Figure 1].

## Trap characterization

Characterization of the imaging optics of the MCG have been extensively described in 28. In this section we report several characteristics that have not been previously reported, principally characteristics of the trapped atoms. To the extent possible, we have attempted to follow established protocols, to facilitate comparison with prior and existing experimental setups.

### Occupancy and loading rate

With the MOT running, loss of an atom from the FORT is most likely by LAC with the next atom to fall into the FORT ^37^. For this reason, the trap occupancy is approximately 50%, with the loading rate being nearly equal to the loss rate, as shown in Figure 3 and Figure 6. The loading rate can be controlled via the overlap of the MOT with the FORT, using the MOT compensation coils to displace the MOT.

### Trap lifetime

**Figure 4.**
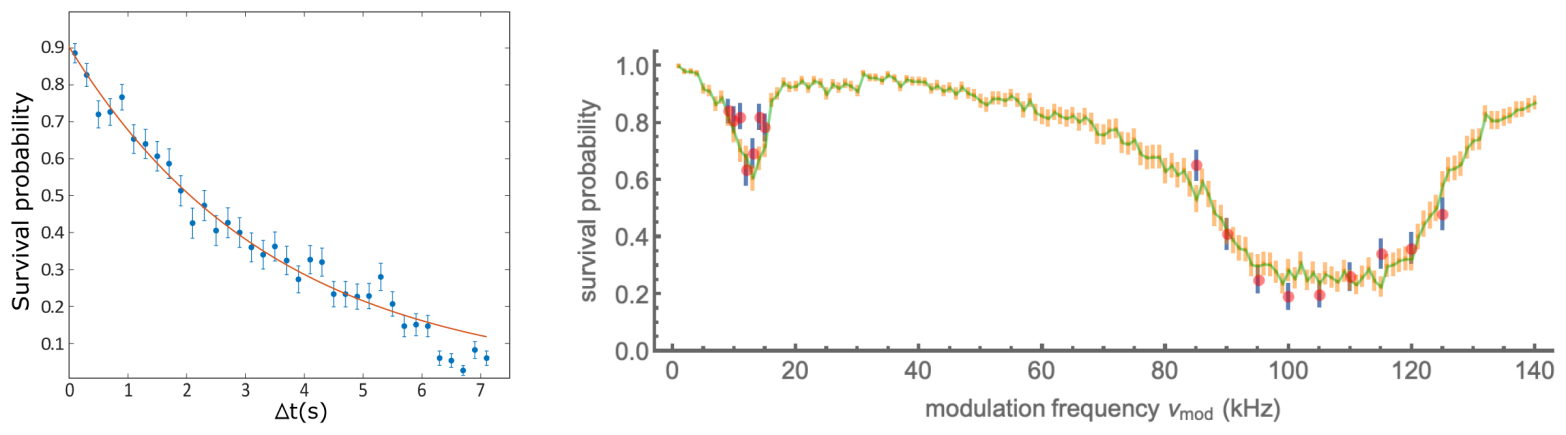
Removal of atoms from the trap with and without parametric excitation. Left: Persistence of a trapped atom in the far-off-resonance trap (FORT) as a function of hold time *∆t*. After detection of an atom by fluorescence, the magneto-optical trap (MOT) beams are turned off and the magnetic field gradient reduced, to prevent capture of a second atom in the FORT. After at time *∆t*, the MOT beams are restored, and the presence or absence of the atom inferred from the fluorescence it produces. Each point shows the average of 150 trials, error bars show ± one standard error assuming binomial statistics. Line shows exponential fit with 1 */e* lifetime 3.5(3) s. See data file Lifetime.csv in *Underlying data* ^35^. Right: Survival probability of an atom in the presence of parametric excitation at modulation frequency *ν*_mod_, as described in the text. Data (simulation) are shown as red (green) points. Error bars indicate ± one standard error assuming binomial statistics. See data file SurvivalVersusModulationFrequency.csv and simulation code file SingleAtomParametricExcitation.jl in *Underlying data* ^35^.

By turning off the MOT beams when an atom’s fluorescence is detected on the APD, it is possible to trap and hold an atom in the FORT without loss by LAC. In this situation atoms can still be lost by collisions with background gas in the vacuum chamber, and by heating from stray light, scattering of the FORT beam, or FORT power or pointing fluctuations. The lifetime of an atom due to these effects was measured, the results of which are presented *Underlying data* ^35^ and shown in Figure 4. The observed lifetime of 3.5(3) s is typical in our setup. The lifetime decreases with increasing pressure in the vacuum chamber, for example when dispensers are heated to release Rb. This suggests that the loss is principally from collisions with background Rb atoms.

### FORT beam waist

The FORT beam waist *w*_FORT_, together with the FORT power, determines the shape of the trapping potential, including the trap depth. Other measurements require knowledge of this trapping potential, e.g. the measurement of temperature by the release-and-recapture method ^38^.

The FORT power can be directly measured outside of the vacuum chamber without great difficulty. *w*_FORT_ can be calculated based on the geometry of the FORT beam outside of the vacuum chamber and the optical properties of the high-NA lens that focuses it. In this strongly-focused scenario, however, *w*_FORT_ is sensitive to aberrations, which could be introduced by the vacuum windows or by the lenses themselves. Such aberrations are difficult to measure, especially *in situ*. Using our input beam diameter and computing the beam waist by gaussian beam optics and the thin-lens approximation, assuming no aberrations, gives the result *w*_FORT_ = 1.2 µm, which we take as a lower limit. Parametric excitation (PE) of the atomic centre of mass motion is a proven method to determine *w*_FORT_ in trapped ensembles. PE heats the ensemble, leading to an observable loss of atoms from the trap ^39, 40^. Although this method has been applied to single trapped atoms ^41– 43^, its interpretation is complicated by the fact that, unlike an ensemble, a single atom does not thermalize. In section **Parametric resonances and trap frequency** we present PE measurements, which by a naïve interpretation imply a *w*_FORT_ = 1.75 µm. A comparison against Monte Carlo (MC) simulation of the PE process, described in section **Parametric resonances and trap frequency**, indicates a value closer to *w*_FORT_ = 1.5 µm, while also suggesting that other factors such as non-parametric heating are important.

Considering the above, *w*_FORT_ is only weakly constrained, to the range 1.2 µm to 1.75 µm. Rather than carry this ambiguity through the rest of the article, we use a nominal value *w*_FORT_ = 1.6 µm for the calculations and measurements in the sections that follow. For this value of *w*_FORT_, the transverse and axial trap frequencies are then $\omega_{\text{r}}=\sqrt{4U_{0}/m_{87}w_{\text{FORT}}^{2}}\approx 56\;\mathrm{kHz}$ and $\omega_{z}=\sqrt{2U_{0}/m_{87}z_{R}^{2}}\approx 6.7\;\text{kHz},$ respectively, where *m*_87_ is the ^87^Rb mass.

### Parametric resonances and trap frequency

Parametric excitation, in which the FORT power is modulated to excite parametric resonances in the atomic motion, is widely used to characterize the trap frequencies in optically-trapped atomic gases ^39, 40^. With ensembles, the heating rate and thus the rate of loss from the trap show resonances at specific frequencies. In the harmonic approximation, these occur at double the trap frequencies, due to the even symmetry of the perturbation to the potential. Corrections due to trap anharmonicity have been studied ^39^ and the technique has been applied to single atoms ^41^.

To measure these parametric resonances we used the following sequence: after loading an atom, we blocked the cooler light, leaving on the FORT and repumper beams, so the atom remained in the now-dark *F* = 2 manifold. We then modulated the FORT power *P*_FORT_ for time *t*_mod_ at a modulation frequency *ν*_mod_ with a depth of modulation of ≈ 20%.

The power modulation was accomplished by sinusoidally modulating amplitude of the radio frequency (RF) voltage that drives the FORT acousto-optic modulator and thus the power of the first diffraction order into a single-mode fibre that leads to the experiment.

Following the trap modulation, we checked for the presence of the atom by turning on again the cooler and collecting fluorescence. We repeated this process for 100 atoms for values of *ν*_mod_ near the second harmonic of the 6.7 kHz longitudinal and 56 kHz transverse trap frequencies predicted for the trap potential with the nominal waist *w*_0_ = 1.6µm. The modulation was maintained for 30 ms in the lower-frequency range and 150 ms in the higher. Results are shown in Figure 4, with resonances at ≈ 12 kHz and ≈ 100 kHz, about 10% lower than expected based on the nominal trap frequencies, although the broad and asymmetric profile of the loss feature makes any frequency assignment imprecise. In the naïve interpretation of the technique, in which the transverse resonance frequency obeys *ω _r_* ∝ $w_{\text{FORT}}^{-1}$ , this would indicate *w*_0_ ≈ 1.75 µm.

To understand better these observations, we studied the PE process by MC simulation, in which atoms drawn from a Boltzmann distribution (see section **Atom temperature**) are allowed to evolve under the modulated potential, Equation 1 with harmonically oscillating *P*_FORT_. These simulations show that the parametric excitation process *per se* is not capable of resonantly heating a single atom out of the trap. This is because the combination of phase-sensitive amplification and trap anharmonicity leads first to an excitation of motion along the resonant axes, phase-shifting due to anharmonicity, and then de-excitation of the same trap motion. This contrasts strongly with the case for trapped ensembles, in which PE plus collisional energy redistribution produces irreversible heating. Nonetheless, the simulations indicate that inclusion of a stochastic element in the PE process can reproduce the main features of the observed survival probability data. To obtain the MC results shown in Figure 4, we included in the dynamics a Langevin term describing isotropic momentum-space diffusion, as would be created by scattering of background light or FORT photons. Adjustment of the simulation parameters “by hand” finds best agreement with a trap waist *w*_FORT_ = 1.47 µm, modulation depth of 30% (22%) and heating rate of 2.5 recoil */*ms (6.5 recoil */*ms) below (above) 30 kHz. It is clear that the accurate interpretation of single-atom PE data is a non-trivial task, and we do not consider this result, absent a fuller characterization of the excitation process, to give a reliable value for *w*_FORT_.

### Atom temperature

**Figure 5.**
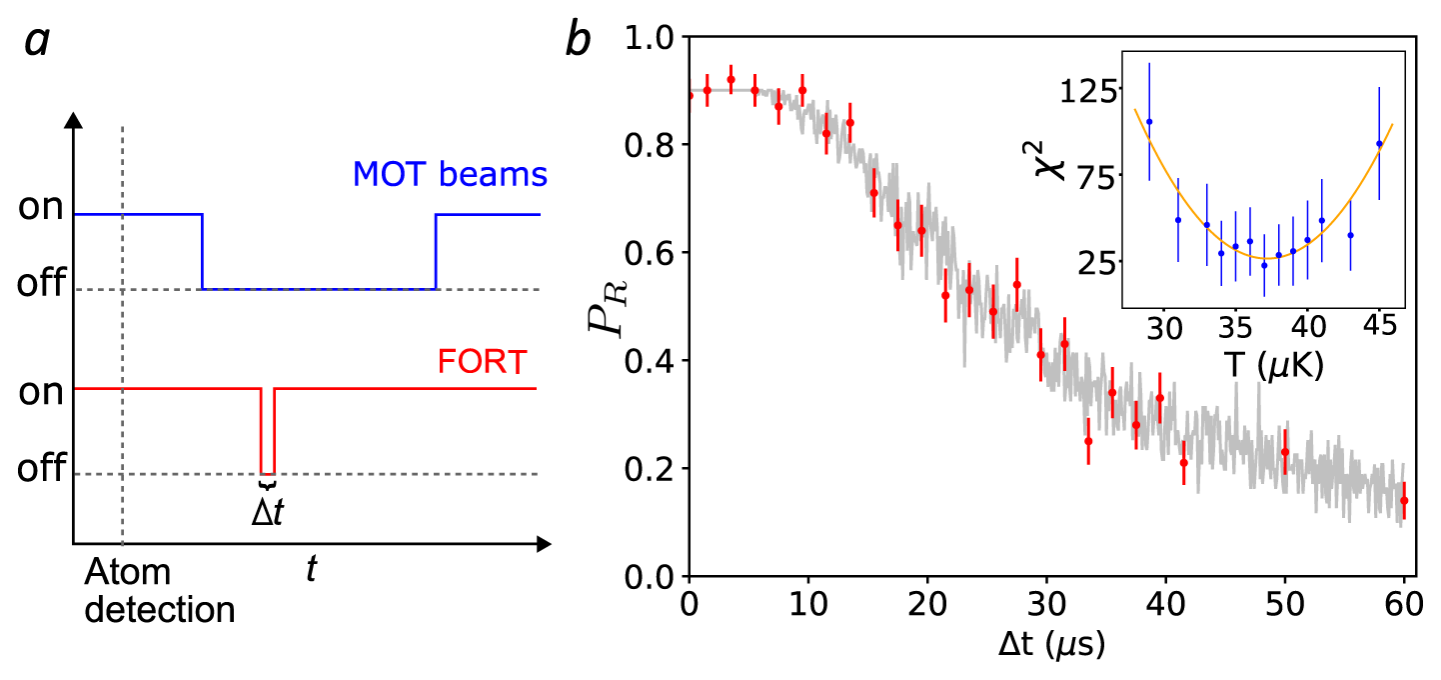
Release and recapture measurement of atom temperature. **a**. Cooling, repumper and far-off-resonance trap (FORT) beams temporal sequence (not to scale). **b**. Observed recaptured fraction *P _R_* as a function of the release time *∆t* (red circles). Each point is the result of 100 trials. Error bars show ± one standard error of *P _R_* assuming a binomial distribution. Grey points show the recapture frequencies observe in a Monte Carlo (MC) simulation with *T*_atom_ = 37 *µ*K, and including a *∆t*-independent 11 percent probability of losing the atom between recapture and fluorescence detection. Inset: *χ* ^2^ distance between data and MC simulation (blue circles) for different temperatures *T*. Error bars show ± one standard error of *χ* ^2^ by propagation of error. A least-squares quadratic fit $\chi_{\text{fit}}^{2}$ ( *T*) (orange curve, see text) finds *T*_atom_ = 37(2) *µ*K. MOT: magneto-optical trap. See Temperature.csv in *Underlying data* ^35^.

We use the release and recapture method to determine the atom’s temperature in the FORT (see *Underlying data* ^35^), as illustrated in Figure 5a. We follow the protocol and analysis described in 38. The MOT and FORT are run until an atom is detected by its resonance fluorescence, as described above. Repumper and cooler beams are then turned off and the MOT magnetic gradient reduced to prevent a second atom from falling into the trap. The FORT is then turned off for a time *∆t*, during which the atom can escape the FORT by ballistic motion. We then turn on the FORT, wait 100 ms and turn on the MOT beams. A recaptured atom is detected by the fluorescence it produces in this last phase. We repeat this sequence 100 times for each value of *∆t*. In Figure 5b we show the recaptured fraction *P _R_* ( *∆t*) for typical conditions.

We compare the experimental observations against a MC simulation of the atom’s probability to be recaptured. In this simulation we assume that, at the moment the FORT is turned off, the atom’s position is gaussian-distributed about the trap center, with zero mean and variances 〈 *∆x* ^2^〉 = 〈 *∆y* ^2^〉 = *k _B_T/*( *m*_87_ $\omega_{r}^{2}$ ) and 〈 *∆z* ^2^〉 = *k _B_T/*( *m*_87_ $\omega_{z}^{2}$ ), which follow from the equipartition theorem under the potential in the harmonic approximation. We assume the atom’s momentum distribution has zero mean and variance $\text{〈}\text{Δ}v_{x,y,z}^{2}\text{〉}=k_{B}T/m_{87},$ which describes the Maxwell-Boltzmann distribution. We then compute the evolved position **x** *_f_* = **x**( *t* = *∆t*) and velocity **v** *_f_* = **v**( *t* = *∆t*) after ballistic flight under gravity for time *∆t*, and the resulting total energy *E _T_* ≡ *m*_87_ ${\text{v}}_{f}^{2}$ /2 + *U*_FORT_( **x** *_f_*) when the FORT is turned on at time *∆t*. If *E _T_ <* 0, the atom is considered recaptured.

For given *T* and *∆t*, we repeat this sequence 100 times to find the recaptured fraction *f _R_* ( *T*, *∆t*). To compare the simulation and experimental results, we calculate *χ* ^2^( *T*) = ∑ _*∆t*_ [ *f*_R_( *T*, *∆t*) − *P _R_* ( *∆t*)] ^2^/ *σ* ^2^( *∆t*), where *σ*( *∆t*) is the standard error of *P _R_* ( *∆t*). As shown in Figure 5b (inset), we compute *χ* ^2^( *T*) for several *T* and fit, by least squares, a quadratic function which we denote $\chi_{\text{fit}}^{2}$ ( *T*). The minimum of $\chi_{\text{fit}}^{2}$ ( *T*) is taken as the best-guess temperature *T*_atom_ = 37(2) *µ*K, with uncertainty ^44^ $\sqrt{2{\left\lfloor \partial^{2}\chi_{\text{fit}}^{2}(T)/\partial^{2}T\right\rfloor }^{-1}},$ where $\left\lfloor \partial^{2}\chi_{\text{fit}}^{2}(T)/\partial^{2}T\right\rfloor$ is the 1 *σ* lower confidence bound on *∂* ^2^ $\chi_{\text{fit}}^{2}$ ( *T*)/ *∂* ^2^ *T*. We note that *T*_atom_ ≪ *U*_0_ */k _B_* ≈ 780 µK, which justifies the harmonic approximation to the trapping potential.

## Collection-efficiency mapping using stochastic loading

**Figure 6.**
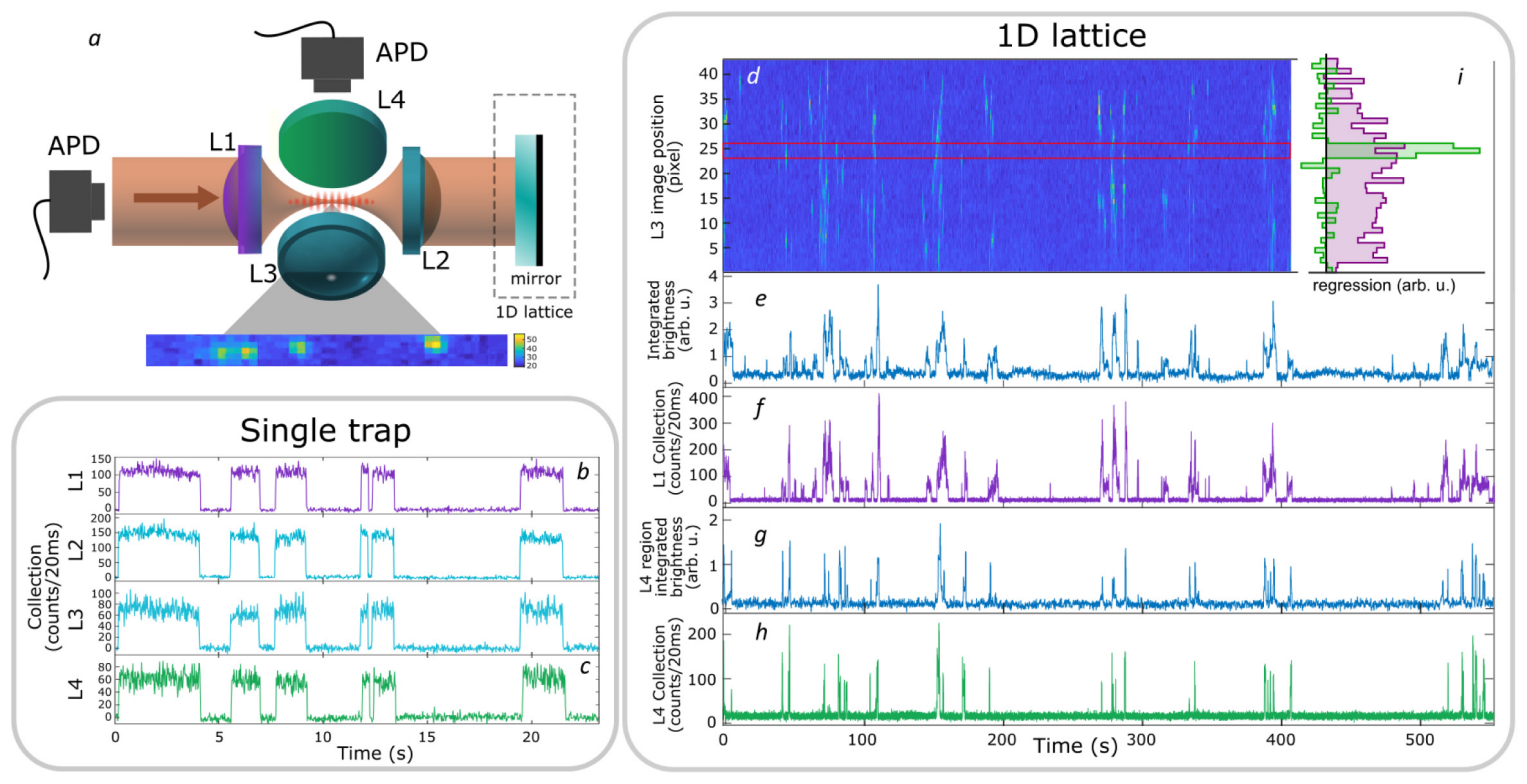
Localized collection of light from a simple far-off-resonance trap (FORT) and a 1D lattice. **a**: Geometry of the trap, collection, and imaging optics. **b** ( **c**): fluorescence signals collected by L1 (L4) of the simple FORT, i.e., with no retro-reflected beam, as seen on avalanche photodiode detectors (APDs). Collection by L2 and L3 lenses is also shown for comparison. The collection efficiency of L3 and L4 is reduced relative to L1 and L2 due to the asymmetric nature of the trap - an atom that moves along the trap axis can leave the region collected by L3 and L4, while remaining in the region collected by L1 and L2. **d**: spatially-resolved fluorescence over time from a continuously-loaded 1D optical lattice, imaged through L3 on a charge-coupled device (CCD) camera. Vertical axis shows axial position in the lattice in pixels, at a magnification of 1 pixel */*µm, horizontal axis shows time of acquisition. Colors indicate fluorescence intensity (arb. u.) integrated over a stripe of transverse dimensionof 3 pixel about the trap axis, increasing from dark to light. The same fluorescence signal is shown integrated over the length of the lattice in **e** and over the pixels between red lines in **g**. **f** ( **h**): Single-mode fluorescence collection by L1 (L4). **i** Contribution of different lattice locations (vertical axis, pixels on same scale as **d**) to the L1 (purple) and L4 (green) signals (horizontal axis). Values determined by linear regression, i.e. least-squares fit of a linear combination of camera pixel signals to the L1 and L4 APD signals (see text). See also RightAngleCollection.csv in *Underlying data* ^35^.

The selectivity in the collection at a right-angle to the trap axis is one of the advantages for the MCG, and provides more access channels when working in the single atom regime. Here we show a correlation-based technique to map the collection of this right-angle access (see *Underlying data* ^35^). We first produce a 1D optical lattice potential by reflecting the FORT light back through lens L2 in order to create a standing wave, as shown in Figure 6a. The input FORT power is reduced to 2.5 mW to partially compensate the intensity boost implied by the standing wave geometry. Atoms were randomly loaded from the free-running MOT into the lattice, and their fluorescence recorded with a camera via lens L3. The camera has a pixel size of 6.45 µm × 6.45 µm, corresponding to 1 µm */*pixel at the atoms. Simultaneously, light collected by L1 (along the lattice axis) and L4 (at a right angle) are coupled into single-mode fibers and detected with APDs.

As shown in Figure 6d, the video records the capture and loss of many atoms at different lattice locations. Spatially- resolved correlation of individual pixels with the L1 and L4 APD signals is then used to measure the spatial distribution of collection efficiency when collecting both along and transverse to the trap axis. Specifically, if *I _i_* ( *t _n_*) is the stripe-averaged intensity at pixel *i* at time *t _n_*, and *R* ^( *L*)^( *t _n_*) is the observed rate of photon detections behind lenses *L* ∈ {L1, L4} at that same time, then a general linear model is

$$R_{C}^{\left(L\right)}(t_{n})=\sum_{i}C_{i}^{\left(L\right)}I_{i}(t_{n})\;(2)$$

where $C_{i}^{\left(L\right)}$ is the time-independent coupling efficiency from pixel *i* to lens *L*. Using the data shown in Figure 6, we find $C_{i}^{\left(L\right)}$ by linear regression, i.e., by minimizing the square error $\sum_{n}{\left[R_{C}^{\left(L\right)}(t_{n})-R^{\left(L\right)}(t_{n})\right]}^{2},$ which is to say we make a least-squares fit with { $C_{i}^{\left(L\right)}$ } as the fit parameters. As expected, and as shown in Figure 6i, the L4 collection is concentrated in a region of FWHM ≈ 2 µm in the camera image, whereas the L1 collection efficiency shows a broad peak spread over many pixels.

For comparison, Figure 6b and c show collection with lenses L1 and L4 with the single trap described in section **System description**. With no optical lattice, collection in the two directions is strongly correlated because each trapped atom explores the entire trap volume, and each channel presents a good signal-to-noise ratio.

## Conclusion

We have described a system for stable, long-term trapping and cooling of single ^87^Rb atoms at the center of a Maltese cross geometry optical system of four high-NA aspheric lenses in vacuum. The system gives high-NA access to the common focal region, which we demonstrate by simultaneously coupling two FORT trapping beams, two single-mode collection fibers, and a high-NA imaging system to observe spatio-temporal atom-number correlations, from which we determine the spatially-resolved single-mode collection efficiencies in the trap-axial and trap-transverse directions. We have studied the principal characteristics of this trapping system, including the loading dynamics, trap lifetime, visibility of single-atom signals, in-trap atom temperature and parametric excitation spectrum. We find trap performance comparable to what has been reported for single-atom traps with one- or two-lens optical systems. We conclude that the multi-directional high-NA access provided by the Maltese cross geometry can be achieved while preserving other trap characteristics such as lifetime, temperature, and trap size.

## Data availability

### Underlying data

Zenodo: Manipulating and measuring single atoms in the Maltese cross geometry - Data.

https://doi.org/10.5281/zenodo.5118863 ^35^.

This project contains the following underlying data:

- HistogramAndNormalizedCrossCorrelation.csv (single atom resonance fluorescence histogram, time series (inset) and normalized cross-correlation in Figure 3).
- Lifetime.csv (removal of atoms from the trap without parametric excitation in Figure 4 (left)).
- SurvivalVersusModulationFrequency.csv (removal of atoms from the trap with parametric excitation in Figure 4 (right)).
- Temperature.csv (release and recapture measurement of atom temperature in Figure 5).
- RightAngleCollection.csv (localized collection of light from a single far-off-resonance trap and a 1D lattice in Figure 6).
- SingleAtomParametricExcitation.jl (Monte Carlo simulation code of the parametric excitation process in Figure 4 (right)).

Data are available under the terms of the Creative Commons Attribution 4.0 International license (CC-BY 4.0).
