# Supplementary material for: Manipulating and measuring single atoms in the Maltese cross geometry
Source: Open Res Eur. 2022 Mar 3;1:102. Originally published 2021 Sep 6. [Version 2] doi: 10.12688/openreseurope.13972.2 (PMC10446080; doi:10.12688/openreseurope.13972.2)
Supplement: Peer review history

# Peer review: Reviewer response for version 1

**DOI:** 10.21956/openreseurope.15058.r27739

**Published:** 2021-10-18

**Authors:** Chi Huan Nguyen, Chang Hoong Chow

**Affiliations:** 1 Centre for Quantum Technologies, National University of Singapore, Singapore, Singapore; 2 Centre for Quantum Technologies, Singapore, Singapore

In this manuscript, Bianchet *et. al *report a detailed study of single neutral atoms trapped in a four-lens arrangement known as the Maltese cross geometry (MCG). In particular, the authors characterize the loading rate, lifetime, and temperature of the single trapped atoms as well as the photon statistics of the fluorescence.

 The four lenses have high numerical apertures allowing four-directional coupling to the single atoms. The setup can therefore obtain strong coupling between single photons and single atoms making it a potential candidate for building up quantum networks. In addition, the MCG system is also useful in experiments that involve disordered ensembles and atomic arrays. In this case, the orthogonal pair of lenses provides the additional selectivity of photon collections at a right-angle to the trap axis.

 Overall, the paper is well written and easy to understand. The figures are produced with high quality. We strongly recommend indexing the manuscript. We have some minor comments and questions:

  

1. The authors presented the microtrap characterization, motivated by the suspicion that the incorporation of a third and fourth lens might have degraded the trapping ability of the system, which is likely not the case for neutral atoms. What is the physical intuition behind this conjecture?
2. We think it is helpful that the authors can provide an estimate of the solid angle covered by four lenses in the manuscript.
3.  As there is not much access to the centre of the 4 lenses on the trapping plane, where is the Rb oven with respect to the trapping zone? Does the atomic beam come from the top or the bottom?
4. As shown in Figure 5.a, there is a delay between the switching on of the FORT and MOT beams. Could the author explain this point in the manuscript?
5. It would be great if the authors could comment more on the background level in the g2 correlation between the collection from L1 and L2. Are they referring to the small offset from 1 at a longer timescale?
6. As shown in the inset of Figure 5.a, the CCD camera image of the atoms trapped in the 1D lattice exhibits a significant tiltness with respect to the camera’s horizon axis. Is it because of the angular misalignment of the optical axes of the two pairs of lenses?
7. When coupling fluorescence from the 1D lattice into a single-mode fiber through L4 lens, does the collection region of FWHM ~ 2um agree with the numerical aperture of the  collection optics?

Is the study design appropriate and does the work have academic merit?

Yes

Is the work clearly and accurately presented and does it cite the current literature?

Yes

If applicable, is the statistical analysis and its interpretation appropriate?

Yes

Are all the source data underlying the results available to ensure full reproducibility?

Yes

Are the conclusions drawn adequately supported by the results?

Yes

Are sufficient details of methods and analysis provided to allow replication by others?

Yes

Reviewer Expertise:

single atom traps, light-matter interaction, ion trap, quantum computing, quantum optics.

We confirm that we have read this submission and believe that we have an appropriate level of expertise to confirm that it is of an acceptable scientific standard.

# Response

**Published:** 2022-02-04

**Authors:** Lorena C Bianchet

**Affiliations:** ICFO, Spain

We thank Drs. Nguyen and Chow for their insightful questions and suggestions.  Specific replies are given below.

*CHN&CHC Point 1: The authors presented the microtrap characterization, motivated by the suspicion that the incorporation of a third and fourth lens might have degraded the trapping ability of the system, which is likely not the case for neutral atoms. What is the physical intuition behind this conjecture?*

**Reply:** The incorporation of a second pair of lenses in the system implies less optical access for the MOT beams, which translates into less alignment freedom, smaller MOT beams and/or more light scattered from the edges of the lenses. There is little published work on very small MOTs, but it is clear that the trapping power of the MOT is a strong function of beam size, so that using extremely small beams to eliminate the possibility of scattered light is not appealing. These same beams act to cool an atom that falls into the FORT. While a reduction in the ~mm size of the MOT beams is not expected to significantly change the optical conditions experienced by an atom in the ~μm sized FORT, an increase in scattered light implies a fluctuating powers and polarizations as seen by the atom. This is the physical intuition behind the conjecture. Other groups have, via personal communications, reported that a system with four high-NA lenses was more difficult to operate than a comparable system with two high-NA lenses.

*CHN&CHC Point 2*: *We think it is helpful that the authors can provide an estimate of the solid angle covered by four lenses in the manuscript.*

**Reply: **We have added an estimate to the revised manuscript.

*CHN&CHC Point 3: As there is not much access to the centre of the 4 lenses on the trapping plane, where is the Rb oven with respect to the trapping zone? Does the atomic beam come from the top or the bottom?*

**Reply:** The MOT is loaded, not from a beam, but rather from the residual Rb vapor in the vacuum chamber.  This is produced by a dispenser located approximately 10 cm below the plane that contains the lenses and other optics shown in Figure 2.

*CHN&CHC Point 4:  As shown in Figure 5.a, there is a delay between the switching on of the FORT and MOT beams. Could the author explain this point in the manuscript?*

**Reply:** This delay is not essential to the technique, but simplifies the data analysis. By including this delay, there is no ambiguity about what fluorescence events are from before the release/recapture modulation of the FORT, and which are from after. It allows us to apply consistently a threshold-based analysis of the observed fluorescence counts to identify recaptured atoms even at short release times.

*CHN&CHC Point 5: It would be great if the authors could comment more on the background level in the g2 correlation between the collection from L1 and L2. Are they referring to the small offset from 1 at a longer timescale?*

**Reply:  **We have expanded the discussion of the g2 correlation function.

*CHN&CHC Point 6: As shown in the inset of Figure 5.a, the CCD camera image of the atoms trapped in the 1D lattice exhibits a significant tiltness with respect to the camera’s horizon axis. Is it because of the angular misalignment of the optical axes of the two pairs of lenses?*

**Reply:** This is simply because of an unintended tilting of the camera that was used to acquire the image. We have added an explanation to the revised article.

*CHN&CHC Point 7: When coupling fluorescence from the 1D lattice into a single-mode fiber through L4 lens, does the collection region of FWHM ~ 2um agree with the numerical aperture of the collection optics?*

**Reply:** We believe the question concerns the width of the green correlation function in Figure 6i, which shows a peak of width  ~ 2μm in the correlation of the L4 signal (counting of photons collected into single-mode fiber through lens L4) and the spatially-resolved camera signal obtained through lens L3.  The width does roughly agree with estimates of the diffraction-limited performance of the optical system. Note that this includes a diffraction-limited width of ~1μm from the collection into single-mode fibre, a diffraction-limited width of ~1μm from the imaging optics onto the CCD camera, and a ~ 1μm broadening due to the 1μm size of the camera pixels.

# Peer review: Reviewer response for version 1

**DOI:** 10.21956/openreseurope.15058.r27548

**Published:** 2021-10-01

**Authors:** Daniel Barredo

**Affiliations:** 1 Laboratoire Charles Fabry, Institut d'Optique Graduate School,CNRS, Université Paris-Saclay, Palaiseau, France

In their manuscript, Bianchet *at al.* report on the characterization of single atom traps using a system of four in-vacuum high NA lenses in a “Maltese cross” geometry. They demonstrate an enhanced light collection efficiency compared to traditional single or dual lens systems, without compromising atom lifetimes, temperatures, or trap sizes.

 Maltese cross geometries offer several advantages for single atom manipulation over dual lens optics. Besides a larger solid angle covered by the four lenses, access to the transverse trapping direction allows for an accurate determination of the size of atomic clouds in disordered ensembles, which is otherwise difficult to estimate, and is crucial for the comparison with theoretical models.  In patterned arrays, the additional right-angle lenses enable individual addressing of the sites in a 1D lattice, and atom-by-atom assembling to obtain defect free arrays. Furthermore, as mentioned in the manuscript, this geometry has been proposed for novel approaches to engineer strong non-classical correlations between photons in combination with single atoms (e.g. Ref. [31]). From this point of view, the experimental setup described and characterized in the present manuscript is very interesting and timely. This setup and some of the results have been already introduced in Ref. [28], but the current manuscript contains additional studies about the performance of the traps with single atoms. Overall, the paper is well written and understandable, even for non-specialists. I therefore recommend indexing.

 There are a few questions/comments that I would like the authors to consider:

1. I believe replacing the word “measuring” in the title by “detecting” or even “imaging” would be more appropriate.
2. In the section describing the MOT, they state “the horizontally-directed beams are of 0.7mm diameter, whereas the vertical beams are of 2.0mm diameter”. Is this the 1/e^2 size?
3. In the measurement of the atom lifetime in the trap (Fig. 4) and of the single atom temperature (Fig. 5), the survival probability is not zero even for the shortest time interval, but rather 0.9 (they mention 11 % loss probability in the caption of Fig. 5).  What is the reason for that? Are these losses related to heating during imaging, for example, or directly associated with the quality of the traps?
4. The authors estimate the trap depth measuring the input power and assuming gaussian optics. This measurement can in principle be done using the atoms as probes, e.g., by spectroscopy to measure induced lightshifts. Have the authors tried this approach?
5. To measure the parametric resonances, the authors pump the atoms in the F=2 manifold and leave the repumper on during the modulation. Do I understand it correctly? If so, why is this necessary?
6. The measured resonances in Fig. 4 are broad. Is it because the modulation is too strong? Have the authors done the experiment with lower modulation depths (< 20 %) to investigate that?
7. Also in Fig. 4, the experimental data are gathered in two different sets at around 12 kHz and 100 kHz. For the low frequency data, they use a modulation time of 30 ms, and for the high frequency range, 150 ms. However, the simulation shows a continuous curve. How is this taken into account in the simulation? I would appreciate more details about the simulation, e.g., if it is a simulation including the three spatial dimensions, or the range of parameters for the Langevin term and the values used.
8. Along the same line, the authors explain that the interpretation of the parametric excitation method is not straightforward for the case of a single atom. Another method usually employed to measure the trapping frequencies (especially the radial one) is to switch the trap off for a few microseconds and on again to excite the radial mode of motion of the atoms in the trap, as it is done, e.g., in Ref [24]. The atoms then oscillate in the trap for a variable time after which one performs a release and recapture experiment. The recapture probability depends on the kinetic energy of the atom and displays oscillations that can be directly mapped onto the trapping frequencies. Possible beatings can then be associated with asymmetries in the trapping potentials. Have the authors explored this method? This can provide additional information to estimate the trap waist.
9. For the 1D lattice the power is reduced from 7 mW to 2.5 mW to “partially compensate the intensity boost implied by the standing wave geometry”. This is still not the expected ratio. Is this mismatch due to losses at the interfaces of the lenses?
10. In Fig 6i the L1 collection efficiency displays a broad peak which is not symmetric. I would appreciate a few words explaining the origin of this asymmetry.
11. Finally, there is a missing word at the end of page 7. “The power modulation was accomplished by sinusoidally modulating amplitude”. I guess it should read “modulating the amplitude”.

Is the study design appropriate and does the work have academic merit?

Yes

Is the work clearly and accurately presented and does it cite the current literature?

Yes

If applicable, is the statistical analysis and its interpretation appropriate?

Yes

Are all the source data underlying the results available to ensure full reproducibility?

Yes

Are the conclusions drawn adequately supported by the results?

Yes

Are sufficient details of methods and analysis provided to allow replication by others?

Yes

Reviewer Expertise:

cold atoms; optical tweezers; single atoms; Rydberg atoms; quantum simulation; quantum optics

I confirm that I have read this submission and believe that I have an appropriate level of expertise to confirm that it is of an acceptable scientific standard.

# Response

**Published:** 2022-02-04

**Authors:** Lorena C Bianchet

**Affiliations:** ICFO, Spain

We thank Dr. Barredo for their insightful questions and suggestions.  Specific replies are given below:

*DB Point 1:  I believe replacing the word “measuring” in the title by “detecting” or even “imaging” would be more appropriate.*

**Reply:** As with any scientific article, it is difficult to capture the exact nature of the work in the few words allowed for a title. In our case, the article describes many different operations on the atoms in the trap, including detection and imaging, but also measurement of the temperature of the atom and atom number statistics, measurement of internal-state dynamics via correlations of resonance fluorescence, the measurement of trap characteristics such as lifetime and trapping beam shape, and manipulations including parametric excitation and lattice confinement. To encompass these many activities, we chose the broad terms “measurement” and “manipulation.” We believe this is an accurate, if not very detailed, title for the work. We also believe that no matter what title we give, the potential reader will have to read the abstract to have a better idea of the content.

*DB Point 2:  In the section describing the MOT, they state “the horizontally-directed beams are of 0.7mm diameter, whereas the vertical beams are of 2.0mm diameter”. Is this the 1/e ^2^ size?*

**Reply:** Yes, it is 1/e ^2^. We have added a note in the revised manuscript to clarify this.

*DB Point 3:  In the measurement of the atom lifetime in the trap (Fig. 4) and of the single atom temperature (Fig. 5), the survival probability is not zero even for the shortest time interval, but rather 0.9 (they mention 11 % loss probability in the caption of Fig. 5).  What is the reason for that? Are these losses related to heating during imaging, for example, or directly associated with the quality of the traps?*

**Reply:** One possible explanation for the loss at small delay Δt is expulsion or heating of the atom in the process of turning off and on the MOT beams. This could arise if, for example, the intensity balance of the six MOT beams is not maintained during turn-off and turn-on.

*DB Point 4:* *The authors estimate the trap depth measuring the input power and assuming gaussian optics. This measurement can in principle be done using the atoms as probes, e.g., by spectroscopy to measure induced lightshifts. Have the authors tried this approach?*

**Reply:** Yes, in fact, we have performed a spectroscopy measurement in order to have a more accurate measurement of the trap depth. The results are in agreement with the estimates made in this article. A proper description of the technique is a bit too involved to include in this article, and is being prepared for publication elsewhere.

*DB Point 5: To measure the parametric resonances, the authors pump the atoms in the F=2 manifold and leave the repumper on during the modulation. Do I understand it correctly? If so, why is this necessary?*

**Reply:** Yes, the repumper was left on in this experiment. With the repumper on, and no cooler light present, the atom will be quickly pumped into the F=2 ground state and remain in that state. The atom’s internal state is not important to the parametric excitation process, because the light shifts are nearly identical for all ground states. Consequently, the repumper or its absence should have no important effect on the parametric excitation by the FORT light. At the same time, our simulations are only able to reproduce the data if there is a significant heating of the atom during the parametric excitation process, beyond what is expected from scattering of the FORT light.  The repumper could potentially contribute to this heating, through off-resonance scattering, or scattering of laser sidebands near the F=2 → F’ resonances.

*DB Point 6: The measured resonances in Fig. 4 are broad. Is it because the modulation is too strong? Have the authors done the experiment with lower modulation depths (< 20 %) to investigate that?*

**Reply:** Yes, we did measure with lower modulation depths. With 1% of modulation depth, the resonance was difficult to see. Between 6% and 20%, we did not observe that the width of the resonance changed significantly.  For this reason, we do not believe that the principal reason for the resonance widths is power broadening.  Rather, we believe it is a consequence of the anharmonicity of the FORT potential.

*DB Point 7: Also in Fig. 4, the experimental data are gathered in two different sets at around 12 kHz and 100 kHz. For the low frequency data, they use a modulation time of 30 ms, and for the high frequency range, 150 ms. However, the simulation shows a continuous curve. How is this taken into account in the simulation? I would appreciate more details about the simulation, e.g., if it is a simulation including the three spatial dimensions, or the range of parameters for the Langevin term and the values used.*

**Reply:** We thank Dr. Barredo especially for this question, which made us aware of a mistake in the article.  Both the experiment and simulations used a modulation time of 150 ms for frequencies ≤ 30 kHz, and of 30 ms for larger frequencies. These times were reversed in version 1, and have been corrected in version 2. In Figure 4, a statistically significant step is in fact visible from 30 kHz to 31 kHz.   The simulation is indeed in three spatial dimensions. The Langevin term, described in the article as “heating rate of 2.5 recoil/ms (6.5 recoil/ms) [for ≤ 30 kHz (> 30 kHz)]” is implemented as an isotropic diffusion in momentum space. The exact calculation method, implemented in Julia, is included in the repository at https://doi.org/10.5281/zenodo.5118863.

*DB Point 8: Along the same line, the authors explain that the interpretation of the parametric excitation method is not straightforward for the case of a single atom. Another method usually employed to measure the trapping frequencies (especially the radial one) is to switch the trap off for a few microseconds and on again to excite the radial mode of motion of the atoms in the trap, as it is done, e.g., in Ref [24]. The atoms then oscillate in the trap for a variable time after which one performs a release and recapture experiment. The recapture probability depends on the kinetic energy of the atom and displays oscillations that can be directly mapped onto the trapping frequencies. Possible beatings can then be associated with asymmetries in the trapping potentials. Have the authors explored this method? This can provide additional information to estimate the trap waist.*

**Reply:** We have not used this method.

*DB Point 9: For the 1D lattice the power is reduced from 7 mW to 2.5 mW to “partially compensate the intensity boost implied by the standing wave geometry”. This is still not the expected ratio. Is this mismatch due to losses at the interfaces of the lenses?*

**Reply:** We estimate the additional optical losses for the retro-reflected beam, i.e., losses incurred in going from the FORT focus to the mirror and back again, to be about 14% (or 1 mW of 7 mW) . This loss is expected to be about equally divided between losses from reflection at lens surfaces and reflection at vacuum window surfaces. In the absence of these losses, retro-reflection (at the same input power) would produce a factor of 4 increase in intensity at the antinodes. With 14% losses, retro-reflection at the same input power would produce a factor of 3.7 increase in intensity. An equal-intensity compensation would thus require 1.9 mW with losses, or 1.75 mW without. By reducing to 2.5 mW, we are thus operating with slightly deeper traps in the lattice case than in the single-pass case.

*DB Point 10: In Fig 6i the L1 collection efficiency displays a broad peak which is not symmetric. I would appreciate a few words explaining the origin of this asymmetry.*

**Reply:** We have added a note in the revised manuscript to clarify this.

*DB Point 11:* *Finally, there is a missing word at the end of page 7. “The power modulation was accomplished by sinusoidally modulating amplitude”. I guess it should read “modulating the amplitude”.*

**Reply:** We have fixed this in the revision.
